# Internal quality control and external quality assurance: a great past opens the way to a bright future

**DOI:** 10.1515/almed-2022-0075

**Published:** 2022-08-17

**Authors:** Mario Plebani

**Affiliations:** Honorary Professor of Clinical Biochemistry and Clinical Molecular Biology, School of Medicine, University of Padova, Padova, Italy

**Keywords:** external quality assurance, internal quality control, quality, total allowable error, uncertainty

This issue of the *Journal* publishes two seminal articles on the evolution and fundamental role of the main tools used by clinical laboratories to evaluate, monitor and improve analytical results: internal quality control (IQC) and external quality assurance (EQA) [1, 2].

These papers should be welcome not only by the readership of the *Journal* but by the whole community of laboratory professionals as it is time to update our knowledge on IQC and EQA, and even more interesting, to better understand if they are still adopted “by tradition” rather than as effective tools for improving analytical quality and reliability of laboratory results. The authors correctly defined the aim of the IQC as “to monitor the examination process in order to avoid producing erroneous information concerning patient health status” [1], and summarized the roots of the IQC with the following sentence: “the oldest model, developed in the 50s was based on a statistical criterion, from calculating the mean of a number of results from a single measurand in the same control sample. It was supported by the use of a control chart that showed the results of control samples plotted in the X-axis vs. time or day on the x-axis”. Thereafter, they moved to describe the significant improvement thanks to the proposal by James Westgard and Coll. of the multi-role algorithm which was implemented in many automatic analyzers [3]. All applications of the IQC, at that time, have been properly defined as statistical process control (SPC), while no consideration was assured to the intended medical use of test results in the design or planning of SPC applications. The development of the concept of “analytical allowable Total Error” and the search for quality requirements which should provide an objective guide to method evaluation and utilization in quality control paved the way to a more “clinical-oriented” vision of the IQC. Finally, the proposals from the 1999 Stockholm Consensus Conference [4], and thereafter from the Milan 1st Strategic Conference of the European Federation of Laboratory Medicine (EFLM) [5] established a well-defined hierarchy of models to set analytical performance specifications (APS) to be used not only to control random errors, but to allow clinical laboratories to check the system alignment to higher-order references [6, 7]. The article recognizes the impressive efforts assured by some eminent scientists of laboratory medicine, but also the role of Scientific Societies and Federations, including the Spanish Society of Laboratory Medicine (SEQC^ML^), which was strongly involved in international initiatives to improve IQC and EQA. In addition, other important advancements have been achieved regarding the “nature” of control materials, namely their “third-party” source and, even more important, their commutability to assure comparability with results obtained in patient sera. However, the scenario is still under evolution as demonstrated by two surveys concerning IQC protocols conducted in 2017 and 2021, respectively and cited in the article. Unfortunately, the results of the two surveys demonstrate a poor awareness by many clinical laboratories of currently recognized requirements of IQC, including the third-party nature of control materials, the use of obsolete operative control rules and the poor appreciation of the importance of the system alignment to higher-order references according to the metrological traceability criteria. Furthermore, a recently published Editorial in* Clin Chem Lab Med* [8] commenting two articles from James Westgard [9] and Mauro Panteghini [10] highlights the need for further efforts to allow laboratory professionals to adopt updated and more accurate IQC programs. In addition, the paper by Carmen Ricòs and colleagues correctly emphasizes the current debate on the moving average quality control and, even more interesting, on the patient-based quality control which should complement and integrate traditional IQC programs [11, 12]. I would like to remind us that the impressive decrease of the analytical errors in the last decades resulted not only from the introduction of automation, optimization of instrumentations and methods, but also from the adoption of IQC and EQC programs which made possible the within-laboratory improvement of analytical quality as well as a valuable inter-laboratory benchmark.

The second article by Carmen Ricòs and Colleagues [2] complements the first one and summarizes the evolution of the several types of external control programs starting from the first program “invented” by Belk and Sunderman in 1947 [13] in order to investigate clinical biochemistry tests and the first program in hematology designed in 1969 by Lewis and Burgess [14]. Although External Quality Assurance (EQA) and Proficiency Testing (PT) programs are both designed to estimate the inaccuracy and imprecision of results between clinical laboratories, in Europe EQA schemes have been always preferred to emphasize their educational nature. However, as reported by the Authors, the coexistence of two types of EQA (mandatory/PT, and education) makes possible for a laboratory participating in both types of programs to receive for the same measurand a report with acceptable performance in one of them but inacceptable in the other, as noticed by Badrick and Stavelin [15]. The authors, when defining EQA, explained the semantic difference between the terms “assessment” and “assurance” which sometimes are used interchangeably: however, the later term should be better used to emphasize that “it is not simply an evaluation of the analytical performance but includes the interpretation of test results and given advice to clinicians about the diagnostic capacity of them” [2]. According to the Authors, the most interesting advancements in EQA programs regard: (a) the nature of control materials, namely their commutability and value assignment; (b) establishment of analytical performance specifications for results evaluation; (c) the introduction of extra-analytical performances evaluation [16, 17]; (d) harmonization among existing EQAs. In particular, Miller and Coll. identified the key factors to evaluate an EQA strength and established six categories [18]: (a) categories 1 and 2 use commutable materials with assigned values by certifies reference methods; category 1 verifies both accuracy and reproducibility, while the second one verifies accuracy only; (b) categories 3 and 4 use commutable materials but do not assign values using certified reference materials; (c) finally categories 5 and 6 use non-commutable and non-reference values control materials. It is clear that clinical laboratories should strive to adopt categories 1 and 2 EQA programs, avoiding participation at least to categories 5 and 6, but initiatives at national and international levels should be performed to allow clinical laboratories to make the right choice, breaking down economic and regional barriers.

The establishment of analytical performance specifications and harmonization among specifications used in EQA is still a nightmare but the pillars for standardization/harmonization have been identified and were summarized by the authors as follows: (1) certified reference materials; (2) reference measurement procedures; (3) recognized reference laboratories; (4) reference interval agreed; (5) comparable EQA programs.

However, it is clear that further improvements in EQA schemes are related to the development and availability of reference measurement procedures and certified (or internationally recognized) reference materials. Otherwise the standardization and metrological traceability of EQA schemes should be available only for 25–30 measurands. Therefore, further efforts should be made to achieve a consensus to better define and improve the quality of EQA schemes for all other measurands.

In summary, the two papers published in this issue of the *Journ*al not only offer the opportunity to update our knowledge on the story of both IQC and EQA, but to rethink our daily practice and to identify current open issues which deserve further improvement initiatives in the near future.

## References

[1] Ricos C, Fernandez-Calle P, Perich C, Westgards JO (2022). Internal quality control – past, present and future trends. Adv Lab Med.

[2] Ricos C, Fernandez-Calle P, Perich C, Sandberg S (2022). External quality control in laboratory medicine. Progress and future. Adv Lab Med.

[3] Westgard JO, Barry PL, Hunt MR, Groth T (1981). A multi-rule She whart chart for quality control in clinical chemistry. Clin Chem.

[4] Fraser CG, Kallner A, Kenny D, Petersen PH (1999). Introduction: strategies to set global quality specifications in laboratory medicine. Scand J Clin Lab Invest.

[5] Sandberg S, Fraser CG, Horvath AR, Jansen R, Jones G, Oosterhuis W (2015). Defining analytical performance specifications: consensus statement from the 1st strategic conference of the European federation of clinical chemistry and laboratory medicine. Clin Chem Lab Med.

[6] Panteghini M (2010). Application of traceability concepts to analytical quality control may reconcile total error with uncertainty of measurement. Clin Chem Lab Med.

[7] Braga F, Pasqualetti S, Aloisio E, Panteghini M (2020). The internal quality control in the traceability era. Clin Chem Lab Med.

[8] Plebani M, Gillery P, Greaves RF, Lackner KJ, Lippi G, Melichar B (2022). Rethinking internal quality control: the time is now. Clin Chem Lab Med.

[9] Westgard JO, Bayat H, Westgard SA (2022). How to evaluate fixed clinical QC limits vs. risk-based SQC strategies. Clin Chem Lab Med.

[10] Panteghini M (2022). Reply to Westgard et al.: ‘Keep your eyes wide as the present now will later be past. Clin Chem Lab Med.

[11] Cembrowski GE, Chandler E, Westgard JO (1984). Assessment of “average of normals” quality control procedures and guidelines for implementation. Am J Clinpathol.

[12] Van Rossum HH (2019). Moving average quality control: principles, practical application andfuture perspectives. Clin Chem Lab Med.

[13] Belk WP, Sunderman FW (1947). A survey of the accuracy of chemical analyses in clinical laboratories. Am J Clinpathol.

[14] Lewis SM, Burgess BJ (1969). Quality control in haematology: report of interlaboratory trials in britain. Br Med J.

[15] Badrick T, Stavelin A (2020). Harmonising EQA schemes the next Frontier: challenging the status quo. Clin Chem Lab Med.

[16] Sciacovelli L, Secchiero S, Padoan A, Plebani M (2018). External quality assessment programs in the context of ISO 15189 accreditation. Clin Chem Lab Med.

[17] Aita A, Sciacovelli L, Plebani M (2018). Extra-analytical quality indicators – where to now?. Clin Chem Lab Med.

[18] Miller WG, Jones GVD, Horowitz L, Weycamp C (2011). Proficiency testing/external quality assessment: current challenges and future directions. Clin Chem.

